# Analysis of Stress Distribution and Displacement Based on the Miniscrew Positions of the Palatal Slope Bone-borne Expander: A Finite Element Study

**DOI:** 10.1055/s-0043-1777823

**Published:** 2024-03-31

**Authors:** Chakree Leeisaramas, Nattapon Chantarapanich, Samroeng Inglam, Kanlaya Insee

**Affiliations:** 1 Division of Orthodontics, Faculty of Dentistry, Thammasat University, Pathum Thani, Thailand; 2 Digital Industrial Design and Manufacturing Research Unit and Department of Mechanical Engineering, Faculty of Engineering at Sriracha, Kasetsart University, Chonburi, Thailand; 3 Division of Oral Diagnostic Sciences, Faculty of Dentistry, Thammasat University, Pathum Thani, Thailand; 4 Thammasat University Research Unit in Dental Biomechanics, Thammasat University, Pathum Thani, Thailand

**Keywords:** palatal slope bone-borne expander, miniscrew positions, stress distribution, nasomaxillary area, finite element

## Abstract

**Objectives** This study aimed to investigate the stress distribution pattern of the palatal slope bone-borne expander on the maxillary area according to a different anteroposterior position of anchored miniscrews using finite element analysis.

**Materials and Methods** Nasomaxillary stereolithography files with three different anteroposterior anchored miniscrew positions of the palatal slope bone-borne expander were determined as model A, B, and C. Each model consists of four supported miniscrews. Model A: two anterior miniscrews were located between the maxillary canine and the first premolar, and two posteriors between the second premolar and the first molar. Model B: two anteriors were between the lateral incisor and the canine, and two posteriors were the same as in model A. Model C: two anteriors were the same as in model A, and two posteriors were distal to the first molar. One turn of expander screws was applied. Maximum principal stress, equivalent elastic strain, equivalent von Mises stress, and transverse displacement were evaluated.

**Results** The maximum principal stress was mostly found at the bone-miniscrew interface. Model A exhibited an intersecting area of stress between the supported miniscrews. The highest value of principal stress was in model B, while model C showed a uniform distribution pattern. The elastic strain pattern was similar to the principal stress in all models. The highest value of equivalent von Mises stress was located on the expander screw. The largest amount of transverse displacement of teeth was in model A, while model C exhibited a more consistent transverse displacement than other models. Vertical displacement of posterior teeth was also noticed.

**Conclusion** Based on the result, it revealed that the various anteroposterior miniscrew placements of the palatal slope bone-borne expander had various patterns of stress distribution and resulted in various outcomes. It may be inferred that model A's miniscrew location was advantageous for obtaining expansion quantities, but model C's miniscrew position was advantageous for maintaining consistent biomechanics.

## Introduction

One typical problem in orthodontic practice is maxillary transverse discrepancy, which affects patients with diverse individual characteristics such as posterior crossbite, crowded teeth, broad buccal corridor, and uneven dental attrition. 1 A technique utilized to address transverse maxillary discrepancy is maxillary expansion, with the objective of optimizing impact of transverse dentofacial orthopaedics while minimizing dentoalveolar adverse effects. 2 Traditionally, conventional tooth-borne palatal expanders are used to expand maxillary arch width; however, adverse effects on anchored teeth may have occurred. 3

In order to create an appropriate posterior occlusion for long-term stability, orthopaedic expansion of the basal bone is essential. 4 5 6 Miniscrew-anchored palatal expanders or bone-borne rapid palatal expanders (B-RPE) have been beneficial to late adolescent or adult patients undergoing maxillary expansion because of their ability to dissipate expansion force directly through basal bone, 7 which reduces undesirable side effects compared to conventional tooth-borne expanders and the need to perform surgery in many cases. 8 Generally, B-RPE is composed of four anchored miniscrews placed at paramedian area or at palatal slope, called palatal slope bone-borne expander, which has been deemed effective and secure regarding anchorage support and success rate. 3 6 9 Even though satisfying outcomes have been achieved clinically using palatal slope bone-borne expanders in some studies, 5 10 11 12 variations in miniscrew positions were addressed, and the mechanical information regarding variation in appliance design, especially anchored miniscrew position, is not sufficient to describe clinical scenario.

Three-dimensional finite element analysis (FEA) is a computerized technique that is used to investigate the impact of mechanical stimuli in biological subjects, and it has been used to investigate the mechanical effect of maxillary expansion. 3 4 6 13 Thus, this study focuses on investigating the stress distribution pattern of palatal slope bone-borne expander on maxillary area according to a different anteroposterior position of anchored miniscrews using FEA that might provide additional mechanical data for explaining possible correlation to clinical outcome of this appliance.

## Materials and Methods

### Three-Dimensional Virtual Nasomaxillary Model and Palatal Slope Bone-Borne Expander

Three-dimensional (3D) nasomaxillary model in this study was acquired from computed tomography (CT) images of an artificial human skull (QS 7/9-E artificial human skull) (SOMSO Modelle GmbH, Coburg, Germany) of Sermboonsang et al studies. 14 15 Dolphin 3D imaging software (Patterson Dental Supply, Chatsworth, United States) was used to build up the model. The outer cortical layer of maxilla was obtained by extracting outer boundary of each maxillary cross-sectional CT image. The aforementioned boundaries were used to build up the 3D stereolithography (STL) exterior model of maxilla. All maxillary tooth models were also segmented to separate from the maxilla model, which included incisors, canines, premolars, and molars. All models generated from CT images were recorded in STL file format. STL models of cortex were subtracted from teeth to build up maxilla with teeth cavity. Then, the cancellous layer was created by 1.6 mm internal offsetting of cortical layer to achieve a cortical bone thickness distribution between 1.2 and 2.0 mm. 16 All STL models, that is, cortex layer, cancellous layer, and teeth, were converted into nonuniform rational basis spline models prior to being completed as computer-aided design (CAD) solid models using CAD software (VISI, Hexagon AB, Stockholm, Sweden).

**Fig. 1:**
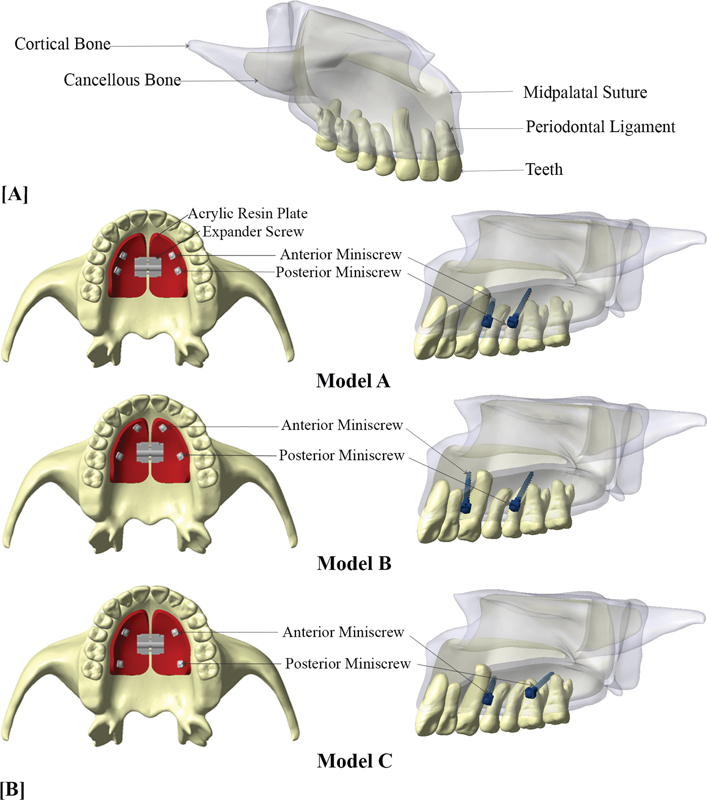
( **A** ) Components of three-dimensional virtual nasomaxillary model. ( **B** ) Three-dimensional virtual nasomaxillary model with palatal slope bone-borne expander in occlusal and sagittal view: Model A, two anterior miniscrews were located between maxillary canine and first premolar, and two posteriors between second premolar and first molar; Model B, two anteriors were located between maxillary lateral incisor and canine, and two posteriors between second premolar and first molar; Model C, two anteriors were located between maxillary canine and first premolar, and two posteriors were distal to first molar.

The cortical layer of maxilla was completed by subtracting the outer boundary of maxilla with cancellous layer. Periodontal ligaments (PDL) were constructed by offsetting tooth root surface by 0.2 mm. 3 The intersection volume between offset model and cortical bone was defined as PDL. Suture was modeled by creating a 0.5 mm width of midpalatal segment. 3 This was performed by separating the CAD model of maxilla in both cortex and cancellous layers using “cut bodies” function. The separated cortex and cancellous layers were then united together as partly ossified one null straight functional unit in approximately posterior one-third of palate geometry to represent the suture, 17 18 and the combined model was assigned material property of suture, stage D maturation. 19 The model was selected to obtain only nasomaxillary area as an area of interest, which facilitates computational calculation and reduces time-consuming process of creating a comprehensive model of entire skull with all of the sutures. 4 The components of geometric nasomaxillary models were displayed in Fig. 1A .

Basically, palatal slope bone-borne expanders consist of a stainless steel jackscrew expander or expansion screw, self-cured acrylic resin plate, and stainless steel miniscrews. Hyrax expanders (Dentaurum, Ispringen, Germany) and miniscrews (Bio-Ray, New Taipei City, Taiwan) were employed as the prototype for modeling. The expander screws have a 0.1 mm transverse widening on each side with every turn of expansion. Expander screw position was determined at the center of the model's palate area between second premolar and first molar, and was linked to four supported miniscrews with 2 mm in diameter and 12 mm in length on acrylic plate.

The palatal slope bone-borne expander appliance was designed in three different anteroposterior miniscrew placement locations, which were designated as model A, B, and C, respectively, and shown in Fig. 1B . Miniscrews were set approximately 8 mm vertically from alveolar ridge 3 and were engaged bicortically. 9 14 Model A: two anterior miniscrews were located between maxillary canine and first premolar, and two posterior miniscrews between second premolar and first molar. 3 12 Model B: two anteriors were located between maxillary lateral incisor and canine, and two posteriors between second premolar and first molar. 6 Model C: two anteriors were located between maxillary canine and first premolar, and two posteriors were distal to first molar. 5 13

### Element Generation

FEA meshing or discretization is the process of transforming a continuous solid region into a discrete computational domain including a finite number of elements that enables the numerical calculation of structural equations using FEA. 20 The assembled geometric models for mathematical analysis were transformed and imported into a FEA pre-processing program (Patran, MSC Software Corp., California, United States) to generate the desired finite element volumetric mesh, prior analysis in FEA program (Marc Mentat, MSC Software Corp., California, United States).

**Fig. 2:**
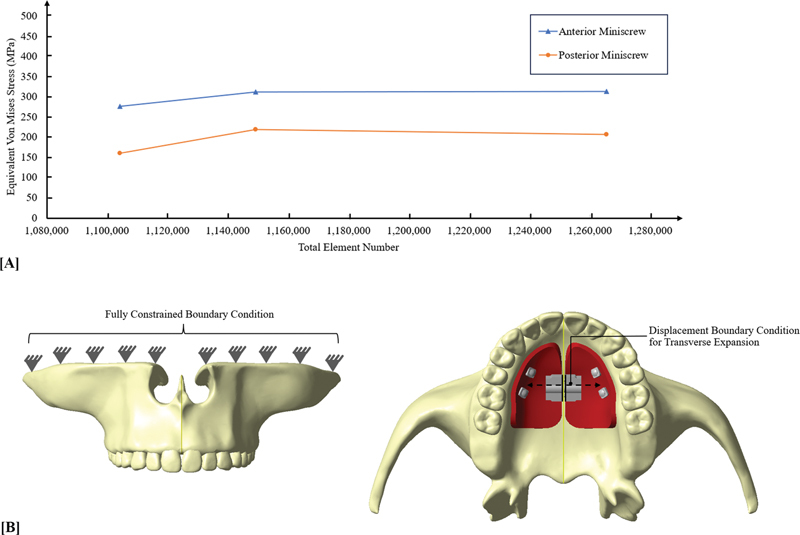
( **A** ) Convergence test and ( **B** ) the boundary condition of a three-dimensional virtual nasomaxillary model with palatal slope bone-borne expander.

Three distinct values of the element were produced, and the corresponding equivalent von Mises stress experienced by miniscrews was examined in the convergence test. According to the convergence results ( Fig. 2A ), a four-node tetrahedral mesh-size of 1.0 mm was used for all portions, except for teeth, PDL, and components of the palatal slope expander, whose 0.5 mm element size was set. Model A consists of 1,278,617 total elements and 364,482 nodes. Model B consists of 1,264,475 total elements and 362,418 nodes. Model C consists of 1,284,717 total elements and 370,408 nodes. The simulated materials were assumed to be elastic, isotropic, and homogeneous, and the model structures were characterized by particular properties listed in Table 1 . 2 3 14 15

**Table 1:** The material properties of each component 2 3 14 15

Component	Elastic modulus (MPa)	Poisson's ratio
Cortical bone	13,700	0.30
Cancellous bone	7,900	0.30
Suture	0.667	0.40
Stainless steel	200,000	0.33
Acrylic resin	2000	0.30
Tooth	20,700	0.30
PDL	0.68	0.49

Abbreviation: PDL, periodontal ligaments.

### Boundary Condition

The boundary condition was displayed in Fig. 2B . The node of geometric model was set to zero displacement and rotation and was confined in the posterosuperior region and in the middle of facial bone at the superior region of zygomatic process of zygomatic bone. 14 15 18

### Contact Condition

All bony parts of geometric model, expander screws, acrylic resin plate, and miniscrews were set as having no relative displacement and assumed that miniscrews and bone contact were fully anchoring. 14 15

### Mechanical Simulation and Parameter Measurement

Simulation of the expansion effect was created by enforced displacement toward the center of expansion screw transversely for 0.1 mm on each side. Maximum principal stress, equivalent elastic strain distribution on the maxilla, von Mises stress on appliance's component, and displacement of teeth were evaluated. The magnitude of each parameter measurement was presented visually using a gradient color column, where a light gray color means the highest value and a deep blue color means the lowest value of each parameter, respectively.

## Results

### Maximum Principal Value of Stress

**Fig. 3:**
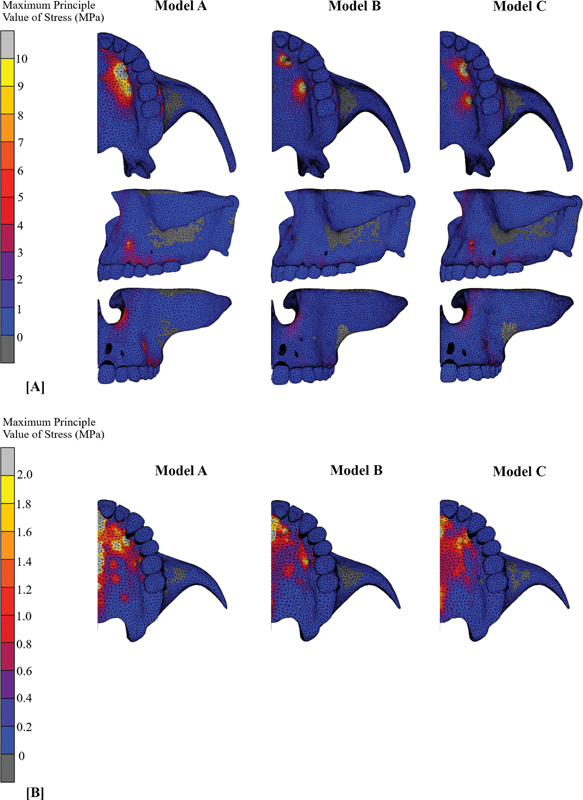
Maximum principal value of stress: ( **A** ) Stress distribution pattern of cortex bone in occlusal, lateral, and frontal view; ( **B** ) Stress distribution pattern of cancellous bone in occlusal view.

In occlusal view, the maximum principal stress of the cortex bone was mostly found at the bone-miniscrew interface for all models (models A–C), and the stress distribution patterns were displayed in Fig. 3A . The stress was not only concentrated at miniscrew-supported area but also noticed the stress accumulation at buccal bone of posterior teeth, lateral wall of nasal cavity, and superior surface of the zygomatic bone, which was obviously seen in lateral and frontal views.

Stress of cortex bone produced by model B had the highest amount with values above 140 MPa, which were observed in areas of two anterior miniscrew engagements, and model A had the lowest amount of stress ( Table 2 ). However, stress in model A apparently showed the intersecting area between anterior and posterior miniscrews and was diffused via palatal vault directed through the midline, with the largest area of stress dispersion along dental arch from canine to first molar. The stress distribution pattern had a uniformly similar pattern in models B and C, which mostly accumulated around anchored miniscrews.

**Table 2:** The maximum principal value of stress of cortical and cancellous bone (MPa)

Model	Maximum principal value of stress of cortical bone (MPa)	Maximum principal value of stress of cancellous bone (MPa)
A	33.28	6.22
B	149.12	5.80
C	48.11	4.97

For all models, stress on cancellous bone was primarily concentrated in the anterior region near incisive foramen and then dissipated through palate in a posterior and lateral direction; however, model A revealed notable area of stress concentration along midpalatal line. Models A and C exhibited the highest and lowest values of stress, respectively; nevertheless, model C showed a more uniform distribution pattern and dispersed evenly all over the palatal contour than other models ( Fig. 3B ).

### Equivalent Elastic Strain

**Fig. 4:**
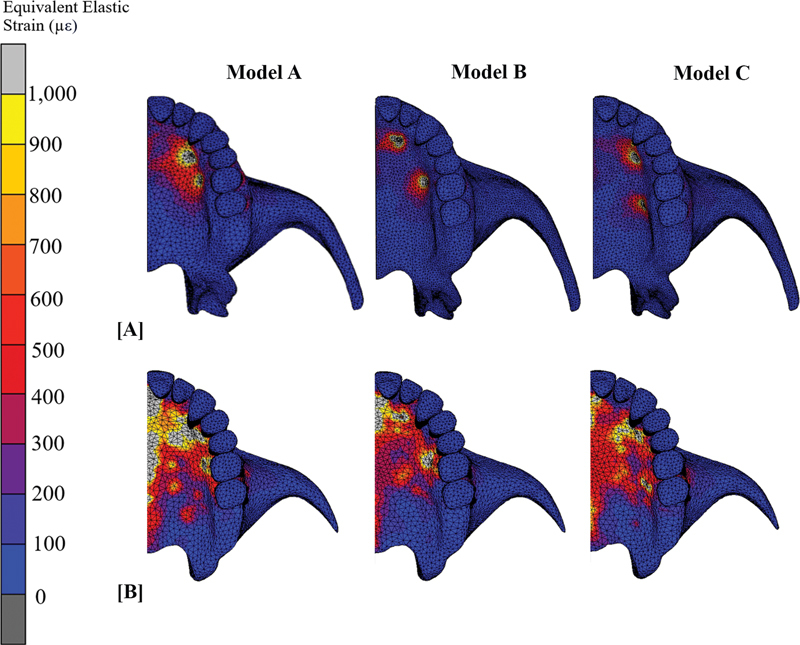
Equivalent elastic strain: ( **A** ) Strain distribution pattern of cortex bone in occlusal view. ( **B** ) Strain distribution pattern of cancellous bone in occlusal view.

The elastic strain value of all models was shown in Table 3 . Equivalent elastic strain distribution pattern was similar to the principal stress pattern for all models. Model A had the lowest value of strain both in cortex and cancellous bone. The highest amount of strain values was 11,222.23, which was detected in areas of two anterior miniscrews of model B and correlated with the highest value of stress in this area, whereas model C has the most consistent strain pattern, which was consonant with the stress distribution pattern ( Fig. 4A , B).

**Table 3:** The equivalent elastic strain of cortical and cancellous bone (µε)

Model	Equivalent elastic strain of cortical bone (µε)	Equivalent elastic strain of cancellous bone (µε)
A	2,733.20	4,476.00
B	11,222.23	8,867.86
C	4,721.58	6,530.13

### Equivalent von Mises Stress

The highest value of equivalent von Mises stress was located on the expander screw for all models. Force was mainly concentrated at expander screw body-acrylic resin plate interface and transmitted via the acrylic resin plate to supported miniscrews. Model A had the highest value of the equivalent von Mises stress at the expander screw, while model B had the lowest value. Anteriorly supported miniscrews had a higher equivalent von Mises stress value than posteriorly supported miniscrews in all models. The value of equivalent von Mises stress on the appliance's component was indicated in Table 4 .

**Table 4:** The equivalent von Mises stress of palatal expander appliance (MPa)

Model	Anterior miniscrews	Posterior miniscrews	Expander screw	Acrylic resin plate
A	468.86	418.81	701.5	202.70
B	397.06	233.71	523.5	108.16
C	445.70	269.42	655.9	460.78

### Displacement

**Fig. 5:**
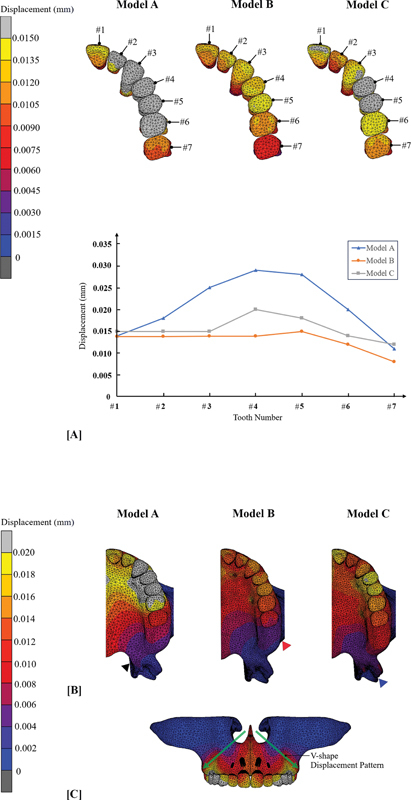
Displacement: ( **A** ) Transverse teeth displacement in occlusal view and graph plot of transverse teeth displacement; ( **B** ) Overall transverse displacement area in occlusal view, pterygomaxillary junction (black arrowhead), maxillary tuberosity (red arrowhead), and pterygoid plate of sphenoid bone (blue arrowhead); ( **C** ) The V-shape (green arrow) transverse displacement pattern of all models in frontal view.

Teeth displacement was principally evaluated in the transverse plane; however, anteroposterior and vertical directions were also evaluated. Model A displayed the greatest amount of transverse displacement of teeth, with a shallow bell-shaped displacement pattern, while model C displayed the least amount of transverse displacement. However, model C displayed a very shallow curve line, which indicates a more consistent transverse displacement pattern when compared to other models, as shown in Fig. 5A . Anteroposterior tooth displacement was only noticed at maxillary central incisor of all models, and the amounts of displacement were 0.003, 0.004, and 0.008 mm in model A, B, and C, respectively. Vertical displacement was mainly found at buccal cusp of posterior teeth. Model A had the largest amount of vertical displacement (0.005 mm), and it was on buccal cusp of the maxillary canine and posterior teeth. The least amount of vertical displacement was in model C (0.002 mm), while model B's vertical displacement (0.003 mm) was nearly equal to that of model C's.

The overall transverse displacement of all models was displayed in Fig. 5B . Model B showed the shortest area of displacement in anteroposterior direction; the displacement was confined posteriorly at maxillary tuberosity. The longest area of displacement was in model C; the displacement pattern dispersed to pterygoid plate of the sphenoid bone, while the area of displacement in model A was close to pterygomaxillary junction. In frontal view ( Fig. 5C ), it exhibited V-shape transverse displacement pattern for all models, where the tip of V was located above vomer bone and the base of the V was located in area of alveolar bone and teeth. This pattern indicated that the more superior the model, the less transverse displacement showed.

## Discussion

This study utilized FEA to primarily investigate and compare stress distribution patterns on the maxillary bone structure with different anteroposterior miniscrew positions of palatal slope bone-borne expander. Several studies 3 4 6 9 17 21 have shown that FEA is a beneficial method for analyzing stress, strain, and force distributions pertinent to orthodontic treatment of maxillary transverse discrepancy. Moreover, FEA is also an alternative, noninvasive, and convenient tool to study how maxillary bone and teeth respond to transverse force from maxillary expander. 22 Within the context of limitations of our study, the findings suggested that there was a variation in stress distribution and outcome pattern depending on the position of the miniscrew. This finding was in the same manner as previous investigations, which was also interested in the effect of miniscrew positions of bone-borne expander. 13

Adult patient skeletal expansion has been demonstrated to be successful when using bone-borne palatal expanders with a variety of appliance designs. 7 8 23 According to available scientific studies, 3 6 10 palatal slope bone-borne expanders had superior advantages over other types of B-RPE because of their versatility in allowing the positioning of anchor screws in a variety of positions, ease of fabrication and adjustment, and less stress accumulation at the area around anchored miniscrews compared to other designs. Expanders can be employed for the purpose of retention once the expansion process has been completed and simply offer direct or indirect absolute anchorage. Despite the fact that B-RPE has been utilized in orthodontic specialty for many years, few studies 6 13 examined the impact of different anchor screw positions of B-RPE on stress distribution, which may be related to treatment outcome.

Many factors, especially the modeling process, affected the stress distribution pattern. It is important in the field of biomechanics to incorporate all contributing factors because reliable results can possibly be obtained if the FEA model reflects the actual skull shape and form. However, the model was partially selected into an interesting nasomaxillary area and simplified some anatomical structures in our study and reduced the time-consuming phase of modeling process. Hence, interpretation of results for clinical application must be approached with caution. The bone thickness of the model may affect stress and strain values. 2 24 Even though a plastic human skull was used as the prototype for reverse engineering in our study, the cortical bone thickness was then modified to achieve a more realistic model. 14 Stress distribution to other areas, not only the palate, was close to what was found by MacGinnis et al 22 ; therefore, a further study including the whole skull with all sutures would demonstrate a more realistic clinical scenario.

The ability of the nasomaxillary model to endure stresses applied during expansion is largely dependent on the material properties and conditions that make up the model. However, there was inconsistent material property value addressed in several studies; it was technically challenging to assign the suitable material properties for each situation, and small changes in these values might not have a meaningful impact on the patterns of stress, strain distribution, and the final displacement results. 25

A report by Boryor et al 4 indicated that stress distribution on maxillary bone is determined primarily by palatal expander design. Different B-RPE designs showed unique stress distribution characteristics. 5 The range of stress values and distribution patterns of the present study coincided with several previous FEA reports. 3 17 22 26 Stress and strain color map gradients or distribution patterns were directly correlated to expansion outcomes. Efficient force transmission to the resistance area as a suture and low stress around the anchorage site are necessary for successful maxillary expansion. 25 Because model A had the lowest value of stress concentration around miniscrews, the residual force was possibly able to transmit along the midline and palatal contour more pronouncedly than in models B and C. 22

The procedure for attaching miniscrews to alveolar bone and acrylic resin connector may affect the force distribution from expander screws through the resin plate and miniscrews. 25 Because there was a difference in the orientation and line of force of the two anterior miniscrews from the expander screw body in model B, this may be the reason why model B had the highest value of force concentration around miniscrews when compared to models A and C. 9 The short distance between each support miniscrew and expander screw of model A may explain why the stress distribution of the cortex bone of model A not only had a unique width with a superimposed area of stress distribution from maxillary canine to second premolar but also exhibited the largest amount of overall transverse displacement. While miniscrews of models B and C had approximately similar distances between anterior and posterior support miniscrews, the stress distribution pattern of cortex bone, where force was mainly concentrated around miniscrews, was fairly indistinguishable.

In addition to the load-bearing region, force also affected buccal bone of maxillary posterior teeth and the distant midface structuress. 22 This could be related to findings from previous clinical studies 6 12 23 which indicated that buccal tooth tipping, buccal alveolar bone height reduction, and aching around the nose area could possibly occur after using a palatal slope bone-borne expander. Interestingly, the stress distribution pattern of cancellous bone was related to the distribution of cortex bone, but in the opposite direction. This circumstance may be because a portion of the energy delivered into the mechanical system by the applied transverse displacement at expander screws was converted into strain energy and compressive strain inside the device's components rather than being transmitted to the sutural and bony structures. 17 Thus, the difference in stress value between cortex and cancellous bone was apparently shown, especially in model B. Anyhow, the stress value of cancellous bone in each model had a little difference in a range of 4 to 6 MPa.

In compliance with the study by Frost, 27 strains valued above 3,000 µε be able to increase the number of microfractures in bone, but the remodeling process normally repairs them, while strains valued above 25,000 µε can cause bone fractures. When stress values and accumulative strain surpass physiological thresholds, the bone remodeling process may enter the pathologic overload phase. This is characterized by a predominance of stress fractures and bone resorption over new bone formation, marginal bone loss, and overstressed and loosening anchored miniscrews. 9 11 17 27 The amount of equivalent elastic strain was approximately between 2,500 and 11,000 µε, which was much smaller than the previous B-RPE FEA study, 14 which exhibited more than 50,000 µε. This may be implied by the fact that palatal slope bone-borne expanders demonstrated less possibility of miniscrews loosening compared to paramedian bone-borne expanders, in accordance with 2014 research conducted by Lee et al. 3

Equivalent von Mises stress is usually used for ductile materials to determine if they will yield or fracture. 2 The study by Lee et al 28 informed us that the cortical bone thickness of the anterior palate is thicker than that of the posterior palate, which may explain why equivalent von Mises stress value at anteriorly supported miniscrews was higher than that at posteriorly supported miniscrews in all models.

The pattern of transverse displacement of both bone and teeth apparently corresponded to the stress distribution pattern. The overall displacement color map indicated that the location of the miniscrews most likely contributed to the suture separation, in addition to the different degrees of ossification the midpalatal suture encountered along its entire length. 17 According to previous studies, 6 12 23 center of resistance and center of rotation of the zygomaticomaxillary complex are present during palatal expansion. The unavoidable V pattern of maxillary halves displacement in frontal view was consistent with previous FEA studies. 14 28 This may be the result of bone splitting at two centers of rotation: one was located at the superior boundary condition of this model or above at frontomaxillary area, and the other was at pterygomaxillary area.

This study was presumed to have isotropic qualities at specific healing time, whereas skull bone is anisotropic and mechanical properties at suture line can be changed over time. The influence of soft tissue effects, bone deformations or remodeling, or individual characteristics such as tooth position and arch form were not taken into consideration in this study. Therefore, these elements should be used in future studies to determine a more accurate scenario. 25 28

## Conclusion

In the scope of this investigation limitations, the study identified differences in stress distribution patterns based on the anteroposterior position of miniscrews in palatal slope bone-borne expander, as determined through computational calculations. In model A, the results suggested favorable miniscrew positions for achieving optimal expansion, including (1) placement sites with a short distance between the anterior and posterior anchored miniscrews to facilitate force transfer from the expander screw, (2) placement sites allowing for a short distance between the expander screw and miniscrews, and (3) placement sites where the orientation of miniscrews aligns with the line of force. On the other hand, in model C, an equally considerable distance between anchored miniscrews and expander screws had an advantage in terms of consistent biomechanics.
